# Carbohydrate restriction uniquely benefits metabolic syndrome and saturated fat metabolism

**DOI:** 10.1186/1753-6561-6-S3-O27

**Published:** 2012-06-01

**Authors:** Jeff S Volek

**Affiliations:** 1 University of Connecticut, USA

## 

Metabolic syndrome (insulin resistance syndrome) represents a group of physiologic signs that indicate a predisposition to obesity, diabetes, and cardiovascular disease. Consistent with the idea that intolerance to carbohydrate is an underlying feature of metabolic syndrome, we present results showing that a low carbohydrate diet results in global improvement in traditional and emerging markers associated with metabolic syndrome. Control diets, restricted in fat, are shown to be less effective. Recent research results mandate a careful re-evaluation of the widespread belief that dietary saturated fat is harmful. Specifically, multiple recent reports find no association between dietary saturated fat intakes and cardiovascular disease (CVD). There is, however, a consistent pattern of increased risk for both CVD and type-2 diabetes associated with increased levels of saturated fatty acids (SFA) in circulating lipids. This raises the important question as to what contributes to increased levels of saturated fat in the blood? Whereas dietary intake of saturated fats and serum levels of SFA show virtually no correlation, an increased intake of carbohydrate is associated with higher levels of circulating SFA. This leads to the paradoxical conclusion that dietary saturated fat is not the problem; rather it’s the over-consumption of carbohydrate relative to the individual’s ability to metabolize glucose without resorting to de novo lipogenesis. From this perspective, insulin resistant states like metabolic syndrome and type-2 diabetes can be viewed as carbohydrate intolerance, in which a high carbohydrate intake translates to increased serum SFA and therefore increased risk. We all stand to benefit, both now and in the future, if a well-formulated low carbohydrate diet becomes an accepted option in promoting health across many sub-groups in our population.

